# Heat-Related Morbidity in Brisbane, Australia: Spatial Variation and Area-Level Predictors

**DOI:** 10.1289/ehp.1307496

**Published:** 2014-04-30

**Authors:** David M. Hondula, Adrian G. Barnett

**Affiliations:** 1 Center for Policy Informatics, Arizona State University, Phoenix, Arizona, USA; 2 Department of Environmental Sciences, University of Virginia, Charlottesville, Virginia, USA; 3 School of Public Health and Social Work and Institute for Heath and Biomedical Innovation, Queensland University of Technology, Brisbane, Queensland, Australia

## Abstract

Background: Extreme heat is a leading weather-related cause of illness and death in many locations across the globe, including subtropical Australia. The possibility of increasingly frequent and severe heat waves warrants continued efforts to reduce this health burden, which could be accomplished by targeting intervention measures toward the most vulnerable communities.

Objectives: We sought to quantify spatial variability in heat-related morbidity in Brisbane, Australia, to highlight regions of the city with the greatest risk. We also aimed to find area-level social and environmental determinants of high risk within Brisbane.

Methods: We used a series of hierarchical Bayesian models to examine city-wide and intracity associations between temperature and morbidity using a 2007–2011 time series of geographically referenced hospital admissions data. The models accounted for long-term time trends, seasonality, and day of week and holiday effects.

Results: On average, a 10°C increase in daily maximum temperature during the summer was associated with a 7.2% increase in hospital admissions (95% CI: 4.7, 9.8%) on the following day. Positive statistically significant relationships between admissions and temperature were found for 16 of the city’s 158 areas; negative relationships were found for 5 areas. High-risk areas were associated with a lack of high income earners and higher population density.

Conclusions: Geographically targeted public health strategies for extreme heat may be effective in Brisbane, because morbidity risk was found to be spatially variable. Emergency responders, health officials, and city planners could focus on short- and long-term intervention measures that reach communities in the city with lower incomes and higher population densities, including reduction of urban heat island effects.

Citation: Hondula DM, Barnett AG. 2014. Heat-related morbidity in Brisbane, Australia: spatial variation and area-level predictors. Environ Health Perspect 122:831–836; http://dx.doi.org/10.1289/ehp.1307496

## Introduction

Extreme temperature events, including single days and extended periods of high and low temperatures, have a negative health impact on urban populations across the globe (e.g., Barnett 2007; Davis et al. 2003; Gabriel and Endlicher 2011; Laaidi et al. 2012; Tong et al. 2010a). A temperature–health association has been demonstrated for major cities across Australia, spanning seasonal temperate climate zones such as Sydney and Melbourne through to Brisbane in the subtropics (Huang et al. 2012; Loughnan et al. 2010; Vaneckova et al. 2010). Improving the resilience of the Australian population to extreme temperatures has been identified as a key research area, particularly in light of the prospects of a warmer climate with more frequent and dangerous heat waves (Bi et al. 2011; Guest et al. 1999; Huang et al. 2011). Researchers have already documented an increase in the number of hot days per year in Australia using meteorological records spanning several decades (Collins et al. 2000), and most climate models project this pattern to continue or accelerate (Guest et al. 1999). Although heat already exerts a toll on the population in terms of increased morbidity and mortality (and decreased comfort and productivity), the impact may be even greater in the coming decades because the aging population will result in more people at high risk. Thus, there is strong motivation to *a*) better understand the risks posed by extreme temperatures, including identifying vulnerable populations and communities, and *b*) increase the capacity of residents and public health officials to take effective action to prevent negative health impacts (Huang et al. 2013).

Brisbane is the third-largest city in Australia, with > 2 million residents. The city has a coastal subtropical climate with warm, humid summers and mild winters. Residents are regularly exposed to high temperatures, and a structural acclimatization that has been used for decades is the characteristic “Queenslander” house that promotes ventilation to capture summer breezes. A trade-off of this house style, however, is that the lack of strong seals and insulation makes it difficult and inefficient to keep houses thermally comfortable during periods of extreme heat. Residents who have access to and are able to afford air conditioning must use greater energy to cool their home than would be required with a different construction style. Thus Brisbane is an interesting city in which to study the health effects of extreme temperatures.

Previous research has found that both high and low temperatures are linked to elevated morbidity and mortality in Brisbane (Bi et al. 2008; Derrick 1965; Huang et al. 2012; Tong et al. 2010b; Wang et al. 2009). However, the impacts across the population are likely to vary among population subgroups. Temperature-related morbidity and mortality are related to exposure, vulnerability, and behavior (Bi et al. 2011). The key variables related to vulnerability are believed to be age (because the human thermoregulatory capacity diminishes over time) and income (because those with more financial resources are likely to have good indoor climate control systems or better insulated housing) (e.g., Tod et al. 2012).

A spatial variation in at-risk individuals across a city would likely result in some areas with higher population sensitivity to temperature than others (Hondula et al. 2012; Reid et al. 2009). There is also spatial variability in temperature exposure because the complexities of the physical and built environment create localized microclimates (Bassil et al. 2009; Rey et al. 2009; Smargiassi et al. 2009). Thus there is strong theoretical support for finding a non-uniform spatial pattern in heat-related morbidity and mortality in Brisbane.

In this study, we explored the spatial variability in sensitivity to heat in Brisbane and identified area-level risk factors associated with the spatial patterns in risk. Risk mapping can help to identify vulnerable areas and create more efficient strategies for resource allocation to mitigate and cope with the effects of extreme weather. Brisbane is an important city in which to conduct this research because it has the potential for significant increases in heat events and heat-related mortality combined with a large and growing population (Bi et al. 2008, 2011; Guest et al. 1999).

## Methods

**Figure 1:**
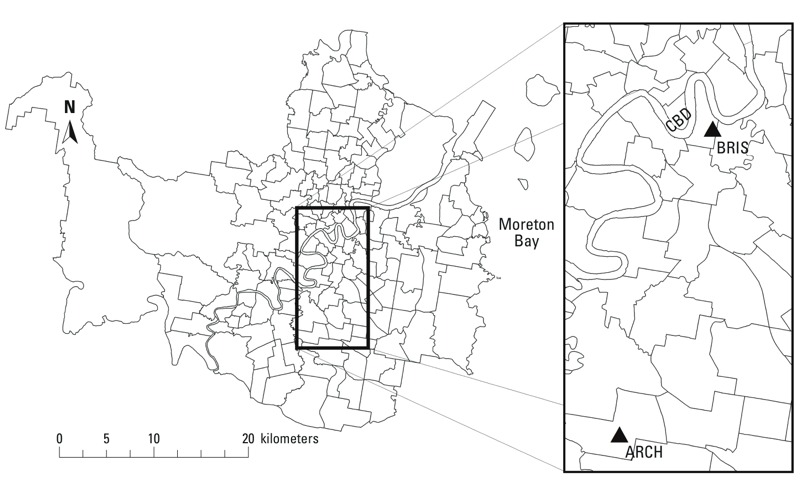
A map of the boundaries of the 158 Statistical Local Areas (SLAs) in the city of Brisbane. The inset shows the central business district (CBD) and the locations of Brisbane (BRIS) and Archerfield (ARCH) weather stations.

**Figure 2:**
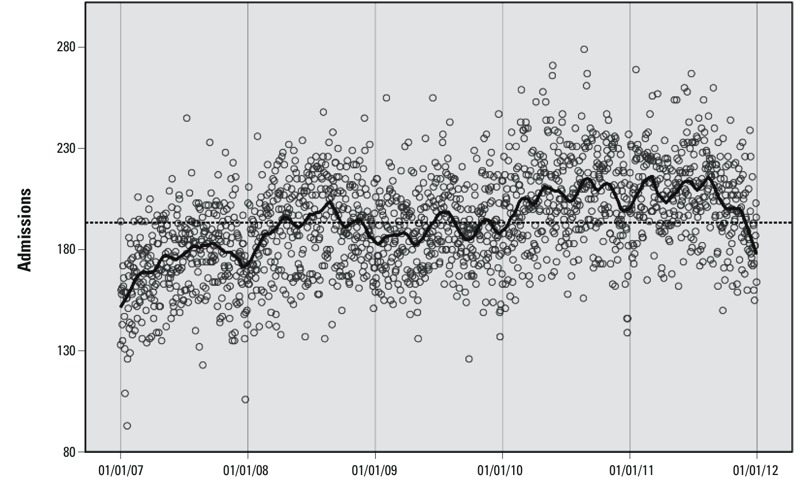
The time series of daily hospital admissions in Brisbane (2007–2011). The dashed line is the mean admissions of 193 per day; the solid black line is a 50-day LOESS moving average used to highlight periodicity that is not evident from the scatter of points.

*Data sources*. We examined morbidity data by using the daily totals of emergency non-accidental hospital admissions for residents of each of the 158 Statistical Local Areas (SLAs, henceforth “areas”) of Brisbane (Figure 1; also see Supplemental Material, Table S1). The data excluded selected external causes, including transport accidents, intentional self-harm, assault, medical complications, legal interventions, and hospital-related adverse events [*International Classification of Diseases, 10th Revision* (ICD-10) codes: V00–V99, X60–X84, X85–Y09, Y35–Y36, Y40–Y84, Y85–Y87.19, Y88–Y89.19]. Admissions were coded by patients’ area of residence. Over the 5 years of data (1 January 2007–31 December 2011) there were 353,231 admissions, with an average of 193 admissions per day. The daily time series shows interannual variability and an increasing mean over time (Figure 2). We used a 50-day local regression smoother (LOESS) for initial visualization of longer-term temporal patterns in the morbidity data (Cleveland and Devlin 1988). Total admissions varied greatly among areas, largely in proportion to population size (see Supplemental Material, Table S1).

Daily weather data were obtained from two stations in the study region, one located near the central business district and another 10 km to the south at Archerfield Airport (Figure 1). These stations were selected because of their continuous record over the study period, with very few missing days (< 0.5%) and their close proximity to populated areas, thus potentially best capturing residential exposure. We used the mean of the two stations’ data on all days for which both data points were available, and the single available measurement for days where one station was missing. The station data were highly correlated (*r* > 0.98). The average annual temperature over the 5-year period was 20.8°C, ranging between 2.5°C and 39.9°C. Mean monthly temperatures peak in January and February above 25°C and are lowest in July near 15°C. The meteorological data were downloaded from the U.S. National Climatic Data Center’s Global Historical Climate Network portal, a compilation of quality-controlled global surface observations (http://www.ncdc.noaa.gov/data-access/land-based-station-data).

Demographic data at an area level were obtained from the Australian Bureau of Statistics 2006 Census Data. From these data we created a set of potential explanatory variables at the area level, including percent of individuals with income < $A150 per week, percent of individuals with income < $A250 per week, percent of individuals with income above $A1,600 per week, population density, percent of structures with two or more stories, percent of individuals requiring public assistance, percent of individuals > 65 years of age, and percent of individuals with no education beyond high school. Variable selection was based on availability of data from the Bureau of Statistics, prior knowledge derived from the heat vulnerability literature (e.g., Reid et al. 2009), and sufficient spatial heterogeneity at the area level for statistical robustness (e.g., there was insufficient spatial variability in race/ethnicity for useful analysis, so these variables were not considered). The total population of Brisbane was 956,130 in 2006. Elevation data were obtained at a 30-m resolution from the Advanced Spaceborne Thermal Emission and Reflection Radiometer (ASTER) Global Digital Elevation Model (GDEM). ASTER GDEM is a product of the Japanese Ministry of Economy, Trade, and Industry (METI) and the U.S. National Aeronautics and Space Administration (NASA). We extracted the mean elevation per area using the Zonal Statistics tool in ESRI ArcMap version 10.0 (ESRI, Redlands, CA, USA).

The admissions data were obtained from Queensland Health. Ethical approval for the study was obtained from the Central Office of the Human Research Ethics Committee, Queensland Health (HREC/12/QHC/39).

*Statistical modeling*. We created a series of statistical models with increasing complexity to estimate the impact of temperature on hospital admission rates and identify the extent to which certain area-level predictors of susceptibility modify the association. We used data from the warm season only, defined as October–March.

The first goal was to estimate the impact of high temperatures on hospital admissions for the whole city (i.e., all areas combined) during the warm season. We used a Poisson regression model in a Bayesian framework using JAGS software version 3.2.0 (http://mcmc-jags.sourceforge.net) to relate citywide admissions to 1-day lagged maximum dry bulb temperature (*tmax1*). Model 1 was

*O_j_ ~ Poisson*(μ*_j_*) log(*μ_j_*) *=* α *+* log(*E*) *+ time_j_ + temperature_j_ time_j_ = hols_j_ + dow_j_ + doy_j_ temperature_j_ =* γ *× tmax1_j_,* [1]

where *O* is the observed number of hospital admissions across Brisbane on day *j*, *E* is an offset to control for population size, and α is the intercept. The offset term *E* is included in model 1 so that results from models 2 and 3 (where *E* varies by area) can be compared with those of model 1. The *time* component comprises categorical variables to account for holidays (*hols*) and day of week (*dow*) and a day-of-year variable (*doy*) used to capture seasonality and long-term trends that are assumed to be unrelated to short-term weather effects. The *doy* variable was created using piecewise basis functions with five knots per warm season or four knots for the warm seasons at the start and end of the study period (Gasparrini et al. 2010).

Four different categories were coded for holidays because exploratory analysis revealed markedly different impacts of holidays on hospital admissions. For example, although many holidays were associated with a reduction in admissions around 15%, Christmas Day was associated with a larger 25% reduction and 2 January was consistently associated with increased admissions.

A linear coefficient γ was chosen for the temperature–hospital admissions relationship (henceforth, “temperature slope”) after a range of other more flexible options were tested (data not shown). We explored using minimum, mean, and apparent temperatures over a range of lags and nonlinear smoothers, and found that a linear 1-day lagged maximum dry bulb temperature (*tmax1*) was most strongly related to daily admissions.

Model 2 was developed to account for both spatial and temporal variability in admissions. The previous model estimated a slope of the temperature–admissions relationship for all Brisbane (γ); here the goal was to estimate the slope in each area using

*O_jk_ ~ Poisson*(μ*_jk_*) log(μ*_jk_*) *=* α*+* log(*E_k_*) *+ time_j_ + temperature_jk_ +* π*_k_ + smooth.area_k_ time_j_ = hols_j_ + dow_j_ + doy_j_ temperature_jk_ =* γ*_k_ × tmax1_j_ smooth.area_k_ = ew_k_ + ns_k_,* [2]

where *smooth.area* is a spatial smoother that accounts for large-scale variability in admissions in the north–south (*ns*) and east–west (*ew*) directions. The spatial smoother was a spline with 2 degrees of freedom for the east–west and north–south effects to allow for the possibility of smooth but nonlinear change in risk across the region. The population size of each area *k* was accounted for with the offset term *E_k_.* Consistent differences between areas were modeled with a random intercept π*_k_*, to account for areas with unusually high or low rates of admissions. (All other variable and subscript definitions can be found in the text following model 1.)

The temperature slope γ*_k_* was modeled as a random effect for each area. Areas with a positive temperature slope and 95% credible interval (CI) excluding zero were considered to have a statistically significant positive association between temperature and hospital admissions, whereas areas with a negative slope and credible interval excluding zero were considered to have an inverse association between temperature and hospitalization. We tested for spatial autocorrelation in the area-level temperature effects using Moran’s *I* statistic (Odland 1988).

Beyond identifying those areas of Brisbane where heat is associated with hospital admissions, we were also interested in identifying factors that predicted the spatial variability in the association with heat. In model 3 we added a linear term λ to modify the heat-slope using area-level variables. Model 3 was specified as

*O_jk_ ~ Poisson*(μ*_jk_*) log(μ*_jk_*) *=* α *+ log*(*E_k_*) *+ time_j_ + temperature_jk_ +* π*_k_ + smooth.area_k_ time_j_ = hols_j_ + dow_j_ + doy_j_ temperature_jk_ =* γ*_k_ × tmax1_j_* γ*_k_ =* γ* + λ *× sla.var_k_ smooth.area_k_ = ew_k_ + ns_k_,* [3]

where γ* is the average temperature coefficient across all areas and *sla.var* is the area-level variable (see full list of variables above in “Data sources”). Positive values for λ mean that the association between heat and hospitalizations is stronger in areas with higher values of the area-level predictor. We explored each of the area-level variables in single-variable regressions, as well as selected combinations in multiple-variable regressions. Candidate models for multiple-variable regression were those in which individual factors demonstrated an association that was statistically significant or approaching statistical significance, and the individual factors were not highly collinear.

In summary, the parameters of interest estimated by the models were γ, the overall estimate of the temperature–admissions slope; γ*_k_*, the estimated temperature effect in each area *k*; and λ, the estimated effect of each area-level variable. We report associations between hospital admissions and a 10°C increase in temperature on the previous day, which is consistent with the difference in temperature between relatively hot and relatively cool summer days.

We used a burn-in and sample size of 5,000 Markov chain Monte Carlo (MCMC) simulations for model 1, and 2,000 for models 2 and 3. Noninformative *N*(0,1000) priors were used for all means and Gamma(1,1) priors for variances.

## Results

*Model 1*. In the first model we estimated the average effect of temperature for Brisbane as a whole. A 10°C increase in temperature on the previous day was associated with a statistically significant 7.2% increase (95% CI: 4.7, 9.8%) in total nonaccidental hospital admissions across the city. This would correspond to 13.9 (95% CI: 9.0, 18.9) additional admissions per day relative to the mean rate of 193 admissions per day.

**Figure 3:**
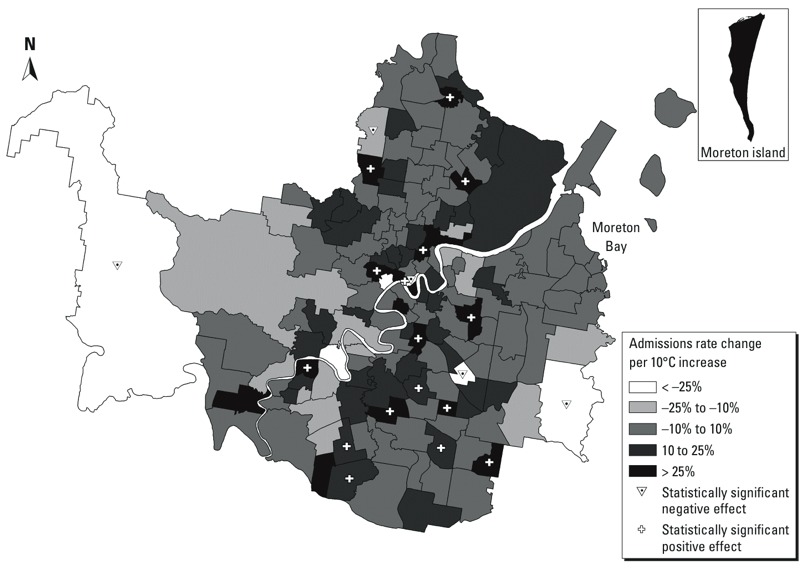
The estimated associations between temperature and hospital admissions for each SLA in Brisbane in terms of the change in daily admissions rate per 10°C increase in temperature on the previous day. White crosses identify areas where the slope is statistically significantly positive.

*Model 2*. In the second model we tested whether the association between temperature and hospital admissions varied across the 158 areas included in the Brisbane study area. We found significant variability, with area-level slopes ranging from a 55% decrease in admissions (95% CI: –75.9, 17.2%) per 10°C increase in temperature to 102% increase (95% CI: 10.6, 257.9%). Statistically significant positive slopes were estimated for 16 areas, and statistically significant negative slopes were estimated for 5 areas (Table 1). Areas with high slopes were geographically spread across the city (Figure 3), and Moran’s *I* statistic indicated no significant spatial autocorrelation in the area-level temperature effect (*I* = –0.02, *p* = 0.19).

**Table 1:** Brisbane areas with statistically significant positive or negative associations between summer temperature and hospitalization on the following day, the magnitude of the association, and area-level characteristics (income and population density) that predict the magnitude of the associations.

SLA name	Heat slope [percent changein admissions per 10°C increasein temperature (95% CI)]	Percent earning > $A1,600/week	Population density (per km^2^)
Median values (all Brisbane SLAs)	4.11	5.95	1670.04
Areas with positive heat slopes
Archerfield	101.71 (10.63, 257.86)	0.52	122.90
Bowen Hills	70.31 (17.60, 148.08)	6.44	926.45
City-Remainder	59.35 (11.21, 129.42)	9.99	2947.13
Deagon	54.80 (15.98, 104.43)	1.77	1169.92
Jindalee	49.22 (13.12, 98.09)	6.50	1958.26
Robertson	38.89 (4.99, 83.50)	5.34	2497.55
Camp Hill	35.05 (11.13, 63.10)	7.99	2138.40
Northgate	34.48 (3.53,77.97)	4.74	1262.11
McDowall	32.95 (2.32, 74.55)	8.52	1574.82
Kuraby	32.49 (2.30, 71.74)	4.39	1434.30
Annerley	28.70 (8.03, 51.45)	4.98	3379.53
Paddington	28.45 (0.63, 62.51)	11.73	3185.38
Inala	24.73 (8.54, 41.97)	0.37	2231.79
Salisbury	23.26 (1.90, 48.40)	1.90	1175.73
Doolandella-Forest Lake	22.28 (5.06, 42.59)	3.46	1757.57
Sunnybank Hills	19.82 (4.71, 36.96)	3.80	2503.21
Areas with negative heat slopes
Burbank	–55.07 (–75.92, –17.22)	9.63	38.56
City-Inner	–43.99 (–64.02, –12.01)	15.66	3870.94
Karana Downs-Lake Manchester	–42.53 (–62.74, –10.89)	6.71	30.97
Mount Gravatt	–29.09 (–43.15, –11.41)	3.89	1127.99
Bridgeman Downs	–23.19 (–39.65, –2.98)	11.62	786.52
The temperature–hospitalization association and area-level modification effects were determined from a hierarchical Bayesian model.			

Areas with the strongest positive associations between temperature and hospitalization tended to be located along a north-to-south–oriented axis through the central business district of the city. Areas with significant associations spanned from near the northern boundary of the study region to the southern boundary. In contrast, there were no areas in the eastern or western thirds of the region with significant associations between temperature and hospitalization. Because the width of the 95% CI varied by area according to sample size, there were some areas with relatively strong associations that were not statistically significant (and vice versa). Areas with significant positive associations often bordered areas where there was no association or an inverse association between temperature and hospitalization.

**Figure 4:**
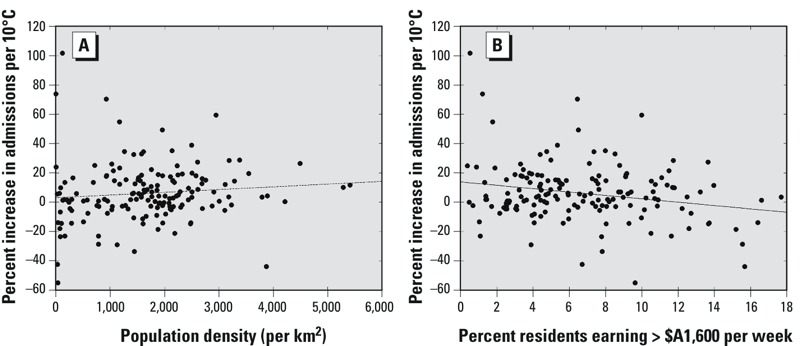
(*A*) The relationship between area-level heat sensitivity, measured as the percent increase in hospital admissions per 10°C increase in temperature on the previous day, and the population density of each area. (*B*) The relationship between temperature sensitivity and the percent of high income-earning residents in each area. The line indicates the best fit using linear regression.

*Model 3*. We next sought to relate the spatial pattern of heat sensitivity (Figure 3) to a number of factors believed to be associated with heat-related risk. Of the eight variables tested, only population density was statistically significantly associated with the spatial pattern of heat sensitivity (Table 2). We estimated a 55.4% (95% CI: 20.7, 93.1%) increase in the temperature slope in association with a 1,000-person increase in residents per square kilometer (Figure 4A). A 1% increase in the proportion of residents with weekly incomes > $A1,600 was associated with an 8.5% decrease (95% CI: –17.4%, 0.7%) in the slope of the temperature–hospitalization association (Figure 4B).

**Table 2:** Estimated percent change in the association between daily maximum temperature (1-day lag) and hospital admissions (λ) per unit change in area-level predictors (model 3).

Predictor	Unit change in predictor	Percent change in temperature–admissions slope (95% CI)
Single area-level predictor models
Percent > 65 years of age	1	–0.1 (–6.9, 6.4)
Percent with income < $A150/week	1	–1.5 (–12.0, 8.8)
Percent with income < $A250/week	1	2.7 (–4.9, 10.2)
Percent with income > $A1,600/week	1	–8.5 (–17.4, 0.7)
Percent with ≤ high school education	10	–1.6 (–29.3, 26.8)
Percent requiring assistance based on disability status	1	1.9 (–19.5, 23.9)
Percent of buildings with ≥ 2 stories	10	0.8 (–17.8, 20.4)
Population density	1,000/km^2^	55.4 (20.7, 93.1)
Multiple area-level predictor model
Percent with income > $A1,600/week (adjusted for population density)	1	–20.3 (–33.3, –7.3)
Population density (adjusted for percent with income > $A1,600/week)	1,000/km^2^	91.1 (46.4, 136.7)
Models were hierarchical, and they accounted for long-term time trends and seasonality in hospital admissions. All models shown included only one predictor with the exception of one multiple variable model that included both income and population density.		

The scatter plots comparing population density and income to heat sensitivity (the temperature slope) for each area show a high level of noise (Figure 4), but these variables were the strongest modifiers of the temperature–hospitalization association. When we included both population density and income in a multivariable regression model, the magnitude of the coefficient increased for both terms, and income became statistically significant. With both terms in the model, we estimated a 91.1% increase in the temperature slope (95% CI: 46.4, 136.7%) for every 1,000-person increase in population density, and a 20.3% decrease in the slope (95% CI: –33.3, –7.3%) for every 1% increase in high earners (Table 2).

## Discussion

The results of this study are summarized by three major findings: *a*) Higher summer temperatures were associated with higher daily hospital admissions on the following day in Brisbane; *b*) statistically significant positive associations between summer heat and hospital admissions were estimated for only some areas of the city, and significant negative associations were estimated for others, so the estimated effect of temperature was spatially heterogeneous; and *c*) on average, areas with greater sensitivity to heat were characterized by higher population densities and lower proportions of high-income residents. Collectively these results provide motivation for continued investment in heat emergency preparedness in Brisbane and offer important insights to help policy makers target the most vulnerable communities. To our knowledge, this study is the first to investigate the within-city variability in sensitivity to heat in Brisbane, a region that presently experiences very high summer temperatures and one predicted to have more extreme heat in the future. The method presented here could be readily applied to other locations where fine-scale geographically referenced health outcome data are available.

Evidence that, on average, hospitalizations increased with elevated temperatures on the previous day is consistent with other studies that have examined relationships between temperature and morbidity (or mortality) in Brisbane (Tong et al. 2010a, 2010b; Wang et al. 2009; Yu et al. 2011). However, we found that a positive statistically significant relationship between temperature and hospital admissions was evident in only some areas of Brisbane. This result suggests that the health burden attributable to heat in Brisbane might be reduced through geographically targeted intervention measures. These strategies might include tree planting, the use of reflective painting on dwellings, and improvements to home insulation and air conditioning. Interestingly, locations of high heat sensitivity were scattered throughout the city; there were only two instances where neighboring areas both had a positive significant slope (Paddington and the northern section of City-Remainder; Inala and Doolandella-Forest Lake). There were also cases where areas with a significant positive slope bordered another with a significant negative slope (City-Remainder and City-Inner; McDowall and Bridgeman Downs).

In total, five areas had a statistically significant negative association between temperature and hospitalization on the following day. It is possible that actions taken by residents of these areas in response to heat may reduce their risk of hospital admission overall. Two of the areas with the strongest negative associations also have very low population densities, and the area with the strongest negative association (Burbank) has ample green space and open water; thus, environmental factors could be behind the protective effect. Finally, the health of residents of these locations may be more negatively impacted by low summer temperatures instead of high ones, which would also lead to the negative statistical relationship we found. Periods of cool, damp conditions during the summer months have been previously associated with elevated respiratory hospital admissions in the United States (e.g., Hondula et al. 2013a).

The result of spatially heterogeneous sensitivity to heat within Brisbane adds to a growing literature reporting non-uniform effects within large metropolitan areas (e.g., Hondula et al. 2012; Smargiassi et al. 2009; Vaneckova et al. 2010). Research in prior decades documenting important between-city differences in sensitivity to heat (e.g., Curriero et al. 2002; Kalkstein and Davis 1989) contributed to the adoption of city-specific heat thresholds, plans, and warning systems (Hondula et al. 2013b; Sheridan and Kalkstein 2004). There is a growing body of research supporting the need to consider within-city differences in sensitivity to heat when developing and implementing municipal emergency response measures and long-term planning efforts. Although it is hard to know the extent to which the results are sensitive to the specific geographic boundaries chosen (the modifiable areal unit problem) (Openshaw 1983), our findings suggest that there are pockets of isolated, vulnerable communities throughout Brisbane that merit targeted efforts to mitigate the negative health impacts of heat. Brisbane public health officials are encouraged to continue investing in heat-health intervention strategies, because the recent data used in this study demonstrate that high temperatures remain a persistent health problem. Reduction of the heat-health burden could be achieved by existing and new intervention strategies tailored to the particular communities where the historical risk has been greatest.

We examined if the spatial pattern in heat sensitivity could be explained by area-level variables. Intervention measures targeting high-risk communities might vary, for example, if the high-risk communities were those associated with lower incomes versus those that were located in places of high building density likely affected by urban heat island effects. Most of the variables examined showed no statistically significant relationship with area-level heat slopes. Most surprisingly, we did not find any evidence that areas with higher percentages of elderly residents had higher hospital admissions during periods of extreme heat. The variables that were related to area-level variability in heat sensitivity were population density and the percent of individuals in each area earning > $A1,600 per week. These two associations are consistent with existing literature. Income has been reported elsewhere as a useful determinant of heat vulnerability (e.g., Hondula et al. 2012; Reid et al. 2009); income is believed to be good proxy for access to air conditioning. Interestingly, we did not find a relationship when examining the percentage of residents within each postal code with income levels associated with poverty status ($A150 and $A250 per week). Instead, we found that areas with higher percentages of high-income earners were at less risk, which could indicate that difficulty coping with heat is not limited to poorer parts of the city. Areas of higher population density are likely to have a higher amount of development and impervious surfaces, thus potentially experiencing urban heat island effects because of the thermal properties of the built environment. We did not find a statistically significant relationship with the percentage of buildings with two or more stories, another variable closely related to the intensity of development and potential urban heat island effects. This contrast might arise because only a small number of areas in Brisbane have numbers of multistory buildings, or because those areas with multistory buildings are also those with many high-income earners.

We acknowledge that this study has several limitations. The most notable limiting factor is sample size; only 5 years of data were available with the area of residence information. Using a relatively recent time period makes the research more relevant for current decision makers, but restricted larger data sets based on more years might have increased our potential to find area-level variability in sensitivity and area-level factors related to spatial heterogeneity. Continued efforts of local, state, and national governments to make health outcome data available to researchers could help alleviate these concerns (and others) in future research. We surmised that areas with higher population density likely experience higher air and surface temperatures during extreme heat events because of urban heat island effects. A comprehensive assessment of the urban heat island of Brisbane does not yet exist and would be helpful in clarifying interactions between the built environment and microclimatic conditions in Brisbane. For example, it may be that suboptimal living conditions (rather than higher temperatures) contribute to higher hospital admissions rates in densely populated areas on hot days. Particular aspects of our methodology that might be addressed differently in future research include the consideration of air pollutants (we did not explore co-exposure effects or potential impacts on the temperature slope) and examination of spatial effects during heat waves [we looked at all summer days and did not treat heat waves differently than isolated hot days (e.g., Ren and Tong 2006)] (Anderson and Bell 2011; Barnett et al. 2012).

## Conclusions

Extreme heat remains a public health concern in Brisbane, as data from the recent period 2007–2011 demonstrate a statistically significant increase in hospital admissions when summer temperatures rise. Our estimates suggest that for every 10°C increase in daily maximum temperature, Brisbane-area hospitals should be prepared for, on average, 14 additional admitted nonaccidental emergency patients on the following day. This 7.2% increase over the daily average of 190 admissions represents a burden on health care and financial resources. Because heat-related illness and death are preventable, continued efforts to protect the public during periods of dangerous heat are warranted. One particular strategy that might be useful in Brisbane is to geographically target various intervention campaigns: We found evidence of statistically significant relationships between heat and hospital admissions in only 16 of the 158 areas of the city. Such geographically targeted efforts should prioritize those places without high-income earners: We found a relationship between area-level income and sensitivity to heat. We also found that areas with higher population density were associated with higher admissions, which may be related to higher thermal stress because of urban heat island effects and/or indoor living conditions. Quantifying within-city variability in sensitivity to heat may be a useful way for researchers to aid public officials in their effort to allocate resources aimed at protecting the public during extreme heat events in an efficient and effective manner.

## Supplemental Material

(124 KB) PDF
