# Supplementary material for: Heat-Related Morbidity in Brisbane, Australia: Spatial Variation and Area-Level Predictors
Source: Environ Health Perspect. 2014 Apr 30;122(8):831–6. doi: 10.1289/ehp.1307496 (PMC4123028; doi:10.1289/ehp.1307496)
Supplement: (124 KB) PDF [file ehp.1307496.s001.pdf]

Environmental Health Perspectives doi:10.1289/ehp.1307496

Supplemental Material

Heat-Related Morbidity in Brisbane, Australia: Spatial Variation and Area-Level Predictors

David M. Hondula and Adrian G. Barnett

Table 1. The names, population, and total admissions during the study period of the 158 Statistical Local Areas in the City of Brisbane explored in this research.

**Statistical Local Area**	**Total Population (2006)**	**Total Admissions (2007-2011)**
Acacia Ridge	6862	4444
Albion	2423	859
Alderley	5072	1771
Algester	8167	3598
Annerley	9566	4014
Anstead	1103	273
Archerfield	581	338
Ascot	4844	1572
Ashgrove	11906	3190
Aspley	11840	5515
Bald Hills	7076	2335
Balmoral	3644	1064
Banyo	4869	2080
Bardon	9111	2375
Bellbowrie	5211	1281
Belmont-Mackenzie	4710	1392
Boondall	8545	3096
Bowen Hills	1584	778
Bracken Ridge	14617	4518
Bridgeman Downs	6882	2056
Brighton	8595	2205
Brookfield (incl. Brisbane Forest Park)	4467	1486
Bulimba	5106	1690
Burbank	1153	253
Calamvale	10178	3844
Camp Hill	9825	3300
Cannon Hill	5355	2062
Carina	9201	3983
Carina Heights	5763	3738
Carindale	13278	2581
Carseldine	6673	3182
Chandler-Capalaba West	1399	290

**Statistical Local Area**	**Total Population (2006)**	**Total Admissions (2007-2011)**
Chapel Hill	9983	2457
Chelmer	2561	826
Chermside	6349	4983
Chermside West	5953	2327
City - Inner	2719	643
City - Remainder	4478	974
Clayfield	9533	3730
Coopers Plains	4090	2292
Coorparoo	13796	4582
Corinda	4456	1984
Darra-Sumner	4054	1644
Deagon	3106	1556
Doolandella-Forest Lake	17505	6097
Durack	6118	3202
Dutton Park	1363	695
East Brisbane	5231	1851
Eight Mile Plains	12021	4047
Ellen Grove	5235	1721
Enoggera	6339	2835
Everton Park	7717	2919
Fairfield	2583	853
Ferny Grove	5488	1572
Fig Tree Pocket	3262	843
Fortitude Valley	5386	2225
Geebung	4105	1956
Graceville	4096	1329
Grange	4041	1216
Greenslopes	8087	3160
Gumdale-Ransome	1531	335
Hamilton	4367	1417
Hawthorne	4311	1159
Hemmant-Lytton	2695	930
Hendra	4113	1186
Herston	1797	1294
Highgate Hill	5429	2049

Statistical Local Area	Total Population (2006)	Total Admissions (2007-2011)
Holland Park	7487	3299
Holland Park West	5520	1961
Inala	13167	7501
Indooroopilly	10731	2605
Jamboree Heights	3278	1006
Jindalee	4988	1662
Kangaroo Point	6869	2642
Karana Downs-Lake Manchester	5710	630
Kedron	11724	4914
Kelvin Grove	4382	2118
Kenmore	8206	2421
Kenmore Hills	2518	784
Keperra	7029	3168
Kuraby	6741	1749
Lota	2980	1015
Lutwyche	2676	1130
MacGregor	5316	1800
Manly	3814	1847
Manly West	10015	1486
Mansfield	10203	2989
McDowall	6901	3417
Middle Park	4051	987
Milton	1729	547
Mitchelton	7033	3299
Moggill	2150	639
Moorooka	9193	4174
Moreton Island	249	36
Morningside	8662	3387
Mount Gravatt	3088	2359
Mount Gravatt East	10248	4180
Mount Ommaney	2202	759
Murarrie	2222	1085
Nathan	1381	387
New Farm	10940	5159
Newmarket	4214	1604

Statistical Local Area	Total Population (2006)	Total Admissions (2007-2011)
Newstead	4820	1231
Norman Park	6753	2125
Northgate	3837	1592
Nudgee	2465	1137
Nundah	8285	3925
Oxley	6378	2715
Paddington	7622	2002
Pallara-Heathwood-Larapinta	1523	695
Parkinson-Drewvale	11861	3748
Pinjarra Hills	413	395
Pinkenba-Eagle Farm	340	207
Pullenvale	3151	787
Red Hill	5271	1911
Richlands	827	574
Riverhills	3955	1092
Robertson	4751	1512
Rochedale	1170	561
Rocklea	1501	947
Runcorn	12475	3951
Salisbury	5524	1663
Sandgate	6504	3135
Seventeen Mile Rocks	8644	2393
Sherwood	4765	3162
South Brisbane	4280	1400
Spring Hill	5236	1639
St Lucia	10610	2695
Stafford	5514	2935
Stafford Heights	6786	2898
Stretton-Karawatha	3806	1002
Sunnybank	7850	3106
Sunnybank Hills	16110	6338
Taigum-Fitzgibbon	7652	3801
Taringa	7489	1724
Tarragindi	9438	3309
The Gap	15665	4406

Statistical Local Area	Total Population (2006)	Total Admissions (2007-2011)
Tingalpa	8887	2589
Toowong	14960	3850
Upper Kedron	2707	724
Upper Mount Gravatt	7910	3798
Virginia	1844	751
Wacol	4452	2054
Wakerley	3903	1336
Wavell Heights	8758	3504
West End	6210	2513
Westlake	4682	992
Willawong	231	123
Wilston	3653	1331
Windsor	6163	2942
Wishart	10268	3466
Woolloongabba	3917	1842
Wooloowin	5485	1896
Wynnum	11719	4661
Wynnum West	10754	4184
Yeerongpilly	2414	913
Yeronga	5357	1994
Zillmere	7464	4257
